# Development of an interaction coding scheme (PaeD-TrICS) to record the triadic communication behaviours in preventive dental consultations with preschool child patients and families: a video-based observational study

**DOI:** 10.1186/s12903-019-0836-z

**Published:** 2019-07-24

**Authors:** Siyang Yuan, Gerry Humphris, Lorna MacPherson, Al Ross, Ruth Freeman

**Affiliations:** 1 Dental Health Services Research Unit, School of Dentistry, University of Dundee, Park Place, Dundee, DD1 4HN UK; 2 Health Psychology, University of St Andrews, St Andrews, UK; 3 Community Oral Health Unit, College of Medical, Veterinary and Life Sciences, University of Glasgow, Glasgow, UK; 4 Public Health, NHS Tayside, Dundee, UK

**Keywords:** Triadic communication, Behaviour coding scheme, Preschool children, Parent, Communication behaviour, Dental consultation, Video observation

## Abstract

**Background:**

There is a paucity of research concerning paediatric dental consultations in primary care. This is potentially due to the difficulty of measuring the communication behaviours in the complex triadic consultations. The present study aims to describe the development and refinement of a coding scheme to record the triadic communication between dental professionals, child patients and parents.

**Methods:**

The PaeD-TrICS was developed from video observation of triadic communications and refined through an iterative process. Its practical applicability was assessed via implementation of the scheme on specialised behavioural coding software. Reliability was calculated using Cohen’s Kappa.

**Results:**

The PaeD-TrICS contains 45 codes. Forty-four dental professional-child-parent communications were successfully coded through administering the scheme on The Observer XT 10.5 system. Cohen’s Kappa was 0.83 (inter-coder) and 0.90 (intra-coder). “Parental verbal facilitation” (mean = 1.68/min) was the most frequent behaviour. Dental professionals’ “dentally engaging talk” (mean = 1.24/min), “praise” (mean = 1.10/min) and “instruction” (mean = 0.62/min) were frequently seen. Children’s common behaviours included “speech other” (mean = 0.66/min) and non-verbal behaviour i.e. “non-verbal agreement” and verbal behaviour “speech yes” (mean = 0.26/min).

**Conclusions:**

The PaeD-TrICS is developed to capture the communication behaviour of the triadic consultations in a preventive dental setting. It demonstrates satisfactory intra- and inter-coder reliability and has been successfully used in paediatric dental consultations.

## Background

Effective communication in paediatric dentistry is of great significance to prepare children to understand dental procedures to enhance oral health outcomes [1, 2]. Central to this, is a clear understanding of the dynamics of triadic communication between dental professional, child patient and parent. The study of triadic communication is limited, as most studies, have tended to concentrate on dyadic (clinician-patient) communication [3, 4]. Despite health professionals being encouraged to adopt child-centred approaches, many factors such as differing styles of parenting and/or limited verbal and non-verbal communication skills may affect effective interaction [5]. This is important, as previous work has shown that only 3 to 14.2% of communications in paediatric appointments can be attributed to children [6–8].

To promote child-centred approaches there is a need to understand the complexities of triadic communications and to achieve this goal it is necessary to have specific and valid tools to capture and measure the communicative behaviours involved in the triadic interaction. Such a tool, in the form of a communication coding system, would enable the quantitative analysis to describe the processes within triadic communication and identify the impact of communication behaviours (including their timing, frequency, and duration) on clinical outcomes. In other words, a detailed coding scheme is required to identify specific triadic communicative strategies that are effective in improving children’s engagement and reducing their distress in the healthcare setting [9].

Dentistry is different from medicine. For example, young children are very prone to becoming anxious when they visit dental practice because of the potentially invasive nature of treatment and perceived threatening stimuli. It is the potential invasive nature, in particular, that distinguishes the dental from the medical setting, and this prospect for child patients can be onerous. Furthermore, it may influence their interactions with dental personnel in the future even with parental presence. Hence dental health providers have a tendency to concentrate more on the child behaviour management skills. These include a number of communication techniques employed when conducting dental procedures such as the “tell-show-do” technique. Frequently this approach is used to inform the child patient of the imminence of a procedure that it will consist of a demonstration before embarking on the procedure. The purpose of the technique is to reduce the child’s anticipatory anxiety and avoid subsequent behavioural problems. Dental professionals’ communication/interaction with children is often restricted to the “tell-show-do” technique or a social interaction such as joking or social “chit chat” in order to build rapport and trust, or to “coax” the child through a negotiation to encourage their acceptance of a particular dental procedure [10]. Furthermore, it is well known that the parent’s presence in the dental consultation has remained a controversial issue in paediatric dentistry [11, 12]. Parent-child interaction behaviours have been grouped into 3 categories: coping-promoting, neutral, and distress-promoting behaviours [13]. Parent coping-promoting behaviours can reduce children’s experience of pain and distress, whereas their distress-promoting behaviours can heighten child pain and distress [14, 15]. It seems problematic, whether or not, parental presence along with their verbal/non-verbal participation will facilitate the dental professional-child interaction and promote child’s cooperation. A coding scheme developed specifically for the paediatric dental setting is therefore essential and ideally should be based on systematic observation within clinical settings.

There are a limited number of paediatric behaviour coding schemes that could be applied in dentistry [10, 16]. Research is limited when restricted to toddlers or pre-schoolers given their limited development and speech ability. A focus on the younger child appears warranted as the early experience of the child in the dental setting can influence the individual when they are older and beyond into their adulthood. Weinstein’s scheme is insufficient on two grounds. First, the system included only the dyadic interaction between a dentist and a child. Second, it was designed to assess behaviour on a macro-level in terms of “granularity” of the communication behaviours [16, 17]. That is, behavioural categories were coded on a frequent sampling basis every 2 s, which lacks specificity and flexibility. Zhou and colleagues’ work has demonstrated the value of a validated system (known as SABICS) in exploring behavioural profiles of Extended Duty Dental Nurses (EDDNs) and children of 3–5-years [18]. It identified the commonly used ‘reassurance’ strategy in the paediatric setting, as one that requires specific attention [18]. The communication strategy of reassurance used by EDDNs was found to command specific caution on its timing of implementation. However this coding scheme was designed for the community setting and investigated especially the dyadic interaction. To our knowledge there were no other studies developing a coding scheme investigating the triadic interaction in a dental setting.

The study aims to describe the development and refinement of a coding scheme to record the triadic interactive behaviours between the dental professional, child patient and parent and to show evidence in supporting the validation of the new coding scheme.

## Methods

### Study setting

The study was conducted within the Scotland’s child oral health improvement programme known as Childsmile [19]. Fluoride varnish application in combination with oral health advice about oral hygiene and sugar consumption/healthy snacking, has been delivered as part of the Childsmile Programme. It is provided by dentists or specially trained EDDNs twice annually from the age of 2 years to 5 years in general dental practice.

### Participants

Five dental professionals with various levels of experience from four General Dental Practices in East of Scotland participated. The professional convenience sample was comprised of three dentists and two EDDNs with one male dentist, the rest being female. The qualified experience of the participating dental professionals covered a wide spectrum. One dentist had over 10 years, another 5 years and one dentist was completing his vocational training. For the two EDDNs, both had over 10 years of qualified experience.

A consecutive convenience sample of 50 child-parent/carer pairs was recruited when they visited dental practice for either a Childsmile appointment or a routine dental check-up with the dentist. Child patients aged between 2 and 5 years, who could speak English with no developmental impairment were eligible. Of these, six observations were excluded later for further video data analysis, due to: (a) two pairs of twins were treated with their twin siblings; (b) one child was the sibling of the participating child and was invited by the parent to receive FV during the video observation; (c) one child was excluded due to observed learning difficulties.

### Study design and data collection

A cross-sectional observational study of Childsmile appointments in a General Dental Practice setting was conducted. Real-time video recording was used to investigate the interaction within a triad of dental professional-child-parent in order to capture both verbal and non-verbal communication behaviours of the interaction. A digital video camera was positioned in the dental surgery room to include in the recorded view all three persons in the triad. The observing researcher (SY) was present throughout to adjust the camera lens for direction and/or focus.

### Development of the Paediatric dental triadic interaction coding scheme (PaeD-TrICS)

*The PaeD-TrICS* was developed to catalogue the triadic interaction in terms of discrete, defined, verbal and non-verbal communication behaviours in a primary dental care setting. The majority of the coding items were drawn from the St Andrews Behaviour Interaction Coding Scheme (SABICS), which was developed to code the Childsmile fluoride varnish interaction between 3 and 5 years old children and the EDDN(s) in a nursery setting [10]. Other codes were added following numerous viewings of the recorded material. The details of the PaeD-TrICS are described in the results section (Table 1).

**Table 1:** Paediatric Dental Triadic Interaction Coding Scheme

Behaviour	Operational definition
Adult behaviour:	
Dental Professional (DP), Parent	
Social talk	DP/Parent’s non-dentally related talk
Information giving	DP/Parent gives oral health/procedure related information.
Information seeking (Questioning)	DP/Parent asks for oral health/procedure related information.
Joke/Humour	DP makes joke/humour on the child that may include a laughter
Child name	DP/parent calls child by name.
Pet name	DP/parent calls child an endearing name
Distraction	DP/parent distracts the child by referring to a toy/painting etc.
Praise	DP/parent makes positive comment on child’s behaviour or attitude
Reassurance	DP/parent describes ease and pleasantness of treatment.
Positive consequence	DP/parent informs child of positive outcome of treatment
Negative consequence	DP/parent informs child of negative/lack of positive outcome if no treatment.
Relate experience	DP relates child’s previous dental experience to the present procedure.
Instruction	DP gives the child instruction to carry out an action
Permission seeking	DP consults child for their consent to carry out an action
Request	DP asks child to carry out an action
Dentally engaging talk	Any talks DP uses to get child engaged in the oral health related talk/treatment
Tell-show-do talk	DP uses tell-show-do technique to instruct child to carry out an action.
Reward	DP promises/gives child a reward, often dependent on behaviour.
Offer for questions	DP offers parent to raise any questions/concerns about child oral health/procedure.
Offer alternative task	DP offers child a lesser challenging task (‘Do you want to sit on mum’s knees?’)
Explanation	DP explains to parent about child uncooperative behaviours which mostly is related to child developmental stage.
Refer to community resources	DP refers to available community resources for parent to access as part of Childsmile procedure.
NV Touch directing	DP physically directs or manoeuvres child’s body, limbs head or mouth.
NV Touch playful	DP touches child with hands, brush, mirror etc. in a playful manner.
NV Touch reassuring	DP/parent uses touch to comfort child.
NV Praise	DP/parent’s nonverbal behaviour to praise/encourage child
NV Procedure demonstration	DP demonstrates to parent/child on dental related procedures (toothbrushing)
Verbal facilitation	Parent helps DP or child to convey information for easier understanding to the third party.
NV Procedure facilitation	Parent physically directs/manoeuvres child’s body to facilitate DP’s procedure.
Child behaviour	
Speech (yes)	Child says ‘yes’
Speech (no)	Child says ‘No’
Speech (other)	Child says any other utterances except for ‘yes’ and ‘no’
Dental talk	Child says anything to reply DP’s oral health related question
Crying/groaning	Verbal sound suggesting pain, fear, upset.
Laugh	Verbal sound suggesting enjoyment.
NV Hide face/mouth	Child covers face with arms or hands.
NV Push away (hand)	Child uses hand/s to push DP or instrument away.
NV Sits up/moves away	Child sits up from lying on the dental chair; stands up (walks way) from sitting.
NV Withdraw	Child withdraws/hides behind/in adult’s body.
NV Agreement	Child conveys acceptance by non-verbal behaviours (nodding head).
NV Shakes head	Child conveys refusal/reluctance/disagreement to information/procedure.
NV Turns head	Child turns head away from DP or a normal position.
NV Interact with instrument	Child holds or touches the instruments (brush, cotton wool, mirror, gloves).
NV Toothbrushing demonstration	Child demonstrates toothbrushing to DP.
NV Pointing	Child points to anything in the surgery to attraction parent’s attention.

**Fig. 1:**
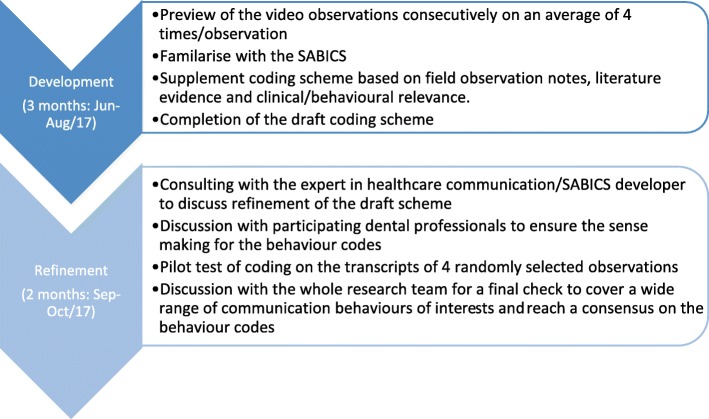
The development and refinement process of the PaeD-TrICS

The process of designing the PaeD-TrICS includes two stages: development and refinement (Fig. 1). The development stage was initiated by watching all of the video recordings four times to familiarise the researcher (SY) with the detailed communication behaviours. Then SY familiarised herself with SABICS to ensure an appreciation of every single code including its operational definitions and specific examples. Meetings with the SABICS development team member were arranged for further clarity of the definitions of codes. For example, differentiating the subtle definitions of the SABICS behavioural codes (e.g. ‘Request’ versus ‘Permission seeking’ and ‘Compliment’ versus ‘Praise’) required further contextual clarification. In addition, SY’s dental professional background, including her non-participatory observation field notes during the recording sessions confirmed her interpretation of the ‘granularity’ of the behaviour codes required at an appropriately specific level for the level of concreteness and flexibility that would be required for analysis and reporting [17]. For instance, SY decided to group the SABICS code ‘compliment’ into a new code ‘social talk’ for inclusion in PaeD-TrICS as in discussion with some members of the research team it was considered that the function of ‘compliment’ served the purpose of dental professional socialising with the child patient. ‘TSD (tell-show-do) talk’ was also included as it is a commonly used behaviour management technique in paediatric dentistry. The inclusion of this specific code enabled the scheme to test whether ‘TSD talk’ will contribute to the prediction of child’s cooperation in receiving dental treatments.

**Fig. 2:**
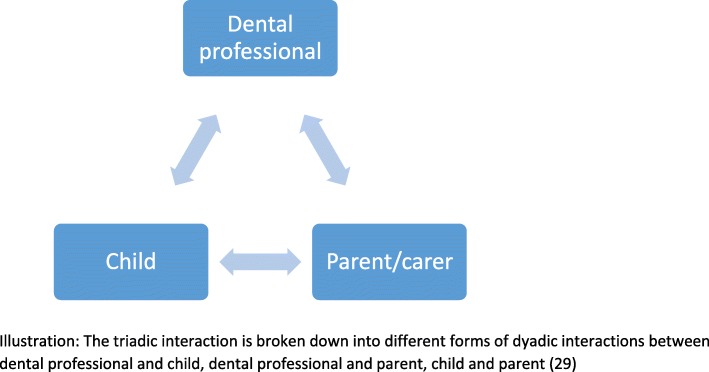
Triadic interaction between dental professional, child patient and parent

Based on an iterative process, the refinement stage was continued in consultation with GH who was involved in the original SABICS development. The draft coding scheme was reviewed, and sample observations were inspected. The coding scheme was also discussed with some of the participating dental professionals to ensure that their professional behaviours identified by the research team matched the staff’s own perception and judgement on patient and parent behaviours. This was not only to reach a consensus on clarifying the overall operational definitions and examples, but also to ensure behaviour codes were sensitive (i) to capture the specifics of the triadic interactive features such as the conversation flow between child and professional, child and parent or professional and parent (Fig. 2), (ii) to embody the typical communicative behaviours that were exhibited in a dental setting and were representative of different participants, (iii) to reflect the specific features of this clinical context. The draft coding scheme was then piloted on four observations, each of these was with a different dental professional. The four video tapes were selected randomly and transcribed verbatim. At the final stage the PaeD-TrICS was discussed with the whole research team to ensure the representation of the full range of potential behaviours of interest before implementing the coding process. A coding scheme with behaviour codes, operational definitions was established (Table 1).

### Description of the PaeD-TrICS

The PaeD-TrICS has been purposively designed to enable both duration and frequency coding. It was developed to code the triadic communication behaviours of a triad of dental professional-child-parent. The PaeD-TrICS contained 45 behavioural codes (Table 1).

All codes are given operational definition with typical examples to illustrate the subtleness for a better understanding. We selected 28 out of 48 codes from SABICS to be kept in the PaeD-TrICS. Given the clinical context of the present study, we included additional behaviours based upon the observations of parents and children’s communication in dental consultations. The dental professionals’ behaviours include those used commonly in managing children’s anxiety such as ‘TSD (tell-show-do) talk’, ‘reassurance’, ‘offer for alternative task’ and those for encouraging child cooperative behaviours such as ‘praise’, ‘reward (stickers)’ and ‘dentally engaging talk’. We did consider carefully children’s limited cognitive and speech ability due to their young age. Hence children’s verbal behaviours were developed simply as ‘speech yes’, ‘speech no’, ‘speech other’, ‘dental talk’, ‘laugh’ and ‘cry’. In contrast to SABICS, we added a new verbal code for children which is ‘dental talk’. This indicates the children’s level of engagement in a dental consultation, which could be used in the future interventions as an outcome measure to indicate children’s involvement in the consultation other than social chit-chat. We included more non-verbal codes for children, most of which could be used to indicate children’s distress behaviours (e.g. ‘withdraw’, ‘pushes away (hand)’, ‘turns head’).

### Coding procedure

The PaeD-TrICS coding scheme was converted to an electronic format for use with Noldus Observer XT (version 10.5). This behaviour software package, enabled us to conduct systematic observation to identify, code and analyse the data according to frequency, duration, timing and sequence of the behaviours. A total of 45 dental professional-child-parent interactions were coded by the primary coder (SY) over a period of 12 weeks. For each interaction, the coding was implemented in steps as presented in Zhou and colleagues’ report [10].

### Calibration and intra/inter-coder reliability check

A second coder (GH) was trained by the primary coder (SY) with a coding manual. Instructions especially regarding the parsing rules (0.5 s as latency) for verbal and non-verbal behaviours were emphasised by SY given the intensive verbal interaction in the dental context. A sample observation with a transcript was provided to the second coder. The sample coding process enabled highlighting the decision rules and improved understanding and agreement on the coding scheme. When discrepancies arose, the second coder referred to the detailed codebook. If necessary, the primary coder (scheme developer) would meet the second coder to resolve any discrepancies that could not be resolved by reading the coding manual containing definitions and examples. Once the calibration process was completed, the second coder started double coding 4 randomly selected videos (10%) from a sample of 44 observations to assess inter-coder agreement.

Cohen’s Kappa with 95% confidence interval estimates was applied to check both inter- and intra-coder reliability for the whole coding scheme [20]. We checked agreement on (i) whether a particular behaviour was present; and (ii) whether behaviours occurred at the same time. The tolerance window was set at 1 s. Both inter- and intra-coder reliabilities were checked twice during a period of 8-week coding period for 44 video observations.

### Ethical considerations

Ethical approval was granted from the East of Scotland Research Ethics Service (Ref: 16/ES/0081). Child and parent participants were consented prior to their Childsmile appointments by SY.

## Results

### Demographic information

Children were aged on an average of 44.9 months (SD = 14.0 months, ranged 24 to 70 months) with 63.6% of children aged older than 3 years. The number of boys (*N* = 21, 47.7%) was comparable with the number of girls. Nearly 82% of the children (*N* = 36) had previously received fluoride varnish. Most children (86.4%) were accompanied by their mothers with four children with fathers, one with both parents and one with the grandparent. The success rate of FVA within 44 children was 79.5%.

### Communication behaviours: duration and frequency

The dental consultation duration ranged from 130 s to 1756 s with an average length of 736 s. The total number of communication behaviours in our sample was 7299. The average number of distinct behaviours of codes assigned per appointment was 161, with 29 behaviours for child patient, 88 behaviours for dental professional and 42 behaviours for parent.

**Fig. 3:**
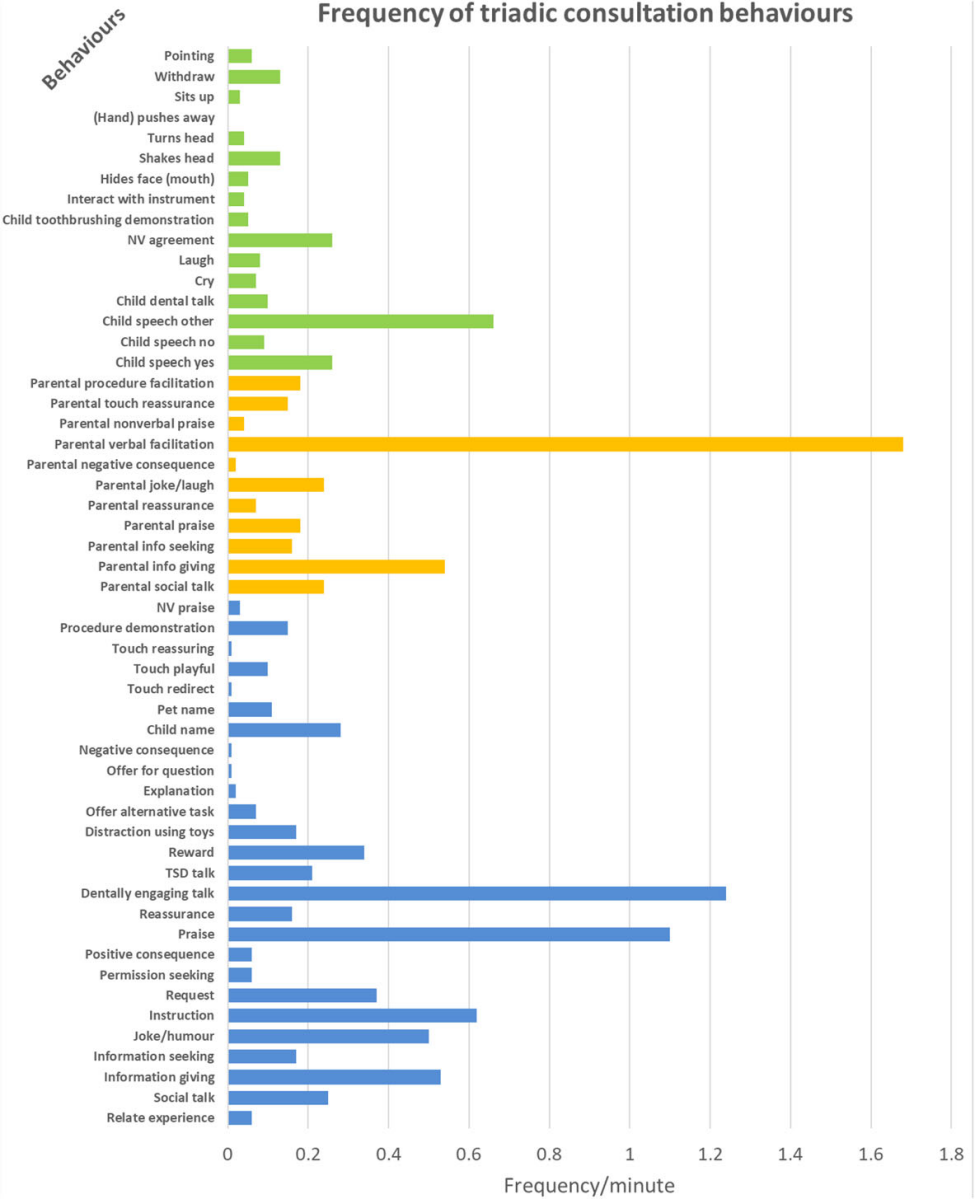
Frequency of triadic consultation behaviours

Given the large variation of the consultation time, we used frequency per minute to adjust differences in the consultation time (Fig. 3). In terms of frequency of behaviours during the triadic consultation, “parental verbal facilitation” (mean = 1.68/min) was the most frequent behaviour during the whole consultation. In regard to dental professionals’ behaviours, “dentally engaging talk” (*n* = 1.24/min), “praise” (*n* = 1.10/min) and “instruction” (*n* = 0.62/min) were mostly frequently seen in the consultation. Children’s common behaviours included “speech other” (*n* = 0.66/min) and non-verbal behaviour i.e. “non-verbal agreement” (and verbal behaviour “speech yes” (*n* = 0.26/min) to express their agreement.

### Intra/inter-coder reliability test

The coding implementation started after we collected all the video tapes to ensure we covered a wide range of important behaviours to be included in the development of the PaeD-TrICS. The primary coder coded all the observations. However, we introduced the 2nd coder to double code four randomly selected videos (nearly 10% of sample) to assess observer agreement and ensure a consistent benchmark. The inter-coder consistency was assessed twice with Cohen’s Kappa of 0.83 (95%CIs: 0.81, 0.85). The intra-coder reliability was checked in the middle of the 12-week coding period to assess drift by comparing ratings of four observations coded by the primary coder (SY). The intra-coder Cohen’s Kappa was 0.90 (95%Cis: 0.89, 0.92). These Cohen’s Kappa values were considered very good according to Altman’s criteria [21].

### Example of coding scheme

Table 2 provides an example of how PaeD-TrICS is applied to code the triadic communication behaviours between the dentist, child and parent to illustrate the participation of each subject in terms of timing and duration of their behaviours.

**Table 2:** Example of how the behaviour codes were applied in a dentist-child-parent interaction

Time stamp	Subject	Behaviour code	Duration (seconds)	Transcript
00:02:51	Dentist	Dentally engaging talk	2.62	Good. And how many times a day do you brush your teeth?
00:02:55	Child	Speech (other)	1.79	Hmn….
00:02:59	Parent	Verbal facilitation	2.12	You think about the right answer
00:03:01	Child	Speech (other)	1.22	Hmn….
00:03:05	Parent	Verbal facilitation	5.04	When do you do it? You do it in the morning….before nursery
00:03:10	Child	Speech (yes)	0.39	Yeah…
00:03:11	Parent	Verbal facilitation	2.00	And then…once before…
00:03:14	Child	Dental talk	0.89	Bedtime
00:03:15	Parent	Praise	0.54	Good!
00:03:15	Parent	Verbal facilitation	1.46	So how many times with that?
00:03:17	Child	Dental talk	0.36	Two
00:03:17	Parent	Verbal facilitation	0.64	Yes!
00:03:18	Dentist	Praise	2.00	That’s really good. Well done.
00:03:21	Dentist	Information giving	3.57	So two times is perfect. That is exactly the right amount.

## Discussion

Communicating with children and parents is of central importance to provide empathetic and child-centred care for children and their parents attending for dental health care [22]. For decades, despite acknowledgement that the paediatric consultation is “triadic”, research evidence has either documented the doctor-parent communication or simply reported child’s conversational contribution by turn-taking [2, 6]. This has not only missed out rich details of communication behaviours in terms of timing, duration and frequency, but also overlooked the significance of documenting the observable behaviours in sequence of all three participants to enable researchers to disentangle the complexity of the triadic interaction process. The new PaeD-TrICS presents a first step to measure and evaluate the clinical significance of these sequential behaviours.

The PaeD-TrICS has demonstrated its capability of embodying the detailed complex process of the triadic interaction in the paediatric dental setting. It included verbal and non-verbal behaviours of all three participants (namely dental professional, child patient and accompanying parent) in a clinical context. Through coding communication behaviours of the three participants in the triad, it allows the investigators to systematically study the relation, content and structure of the triadic consultation compared with previous research [8, 23, 24]. In addition, the results showed that codes included in the observation system can be applied in a consistent approach with satisfactory intra- and inter-coder reliability. Furthermore, with the application of the video observation software (i.e. Observer XT), the new coding scheme not only enables researchers to discover the sequence, duration and frequency of communication behaviours, but also makes it possible to examine the effect of communication behaviours on outcomes (clinical outcome, behaviour outcome etc.) and investigate the association between behaviours in the forms of turn-taking.

Echoed with our statement on utility of the new coding scheme, the current findings from the video observation has indicated that the consultation time varied greatly from just over 2 min to nearly 30 min. In terms of turn-taking by verbal and non-verbal behaviours, children’s participation on average is comprised of 18% of the total consultation, which is slightly higher than previously reported data [2]. Regarding the large variation of the consultation time, we are able to tease out the most frequent communication behaviours by different participants by using the frequency of behaviour per minute. In this study, “parental verbal facilitation” and “information giving” are the most frequent adult behaviours. It is worth noting that “information giving” included dental professional passing the oral health information to the parent as well as the parent informing the professional of the child’s medical and/or dental conditions. Given the nature of the Childsmile appointment, part of the dental professionals’ task is to provide oral health advice to parents regarding fluoride usage, oral hygiene and diet, which explains the frequent use of “information giving” behaviour. The high usage of “parental verbal facilitation” found in our study is inconsistent with previous study in which parents assumed the executive power over the child and managed information given to their children [26]. Instead, in our study parents maintained a supportive role in facilitating the information passed from dental professionals to children by tailoring it in a child-oriented manner. Part of the explanation might be (i) parenting styles have been shifted from authoritarian to supportive styles; (ii) our study encounter is prevention focussed and questions from dental professionals directed to children are highly related to daily lifestyle such as toothbrushing and snacking routines. Both adults in the triadic consultation believe that children are able to answer such questions on their own, therefore parents are more likely to provide their ‘interpreting’ assistance in translating the questions for their child. Consequently, we found that children’s involvement in the consultation is mostly seen in the form of verbal contribution (i.e. ‘speech other’ and ‘speech yes’) and non-verbal participation (i.e. “non-verbal agreement”). When looking at dental professionals’ behaviours, ‘dentally engaging talk’ and ‘instruction’ are the most common behaviours in the triadic consultation. This is in line with previous findings that found ‘dental-oriented communication to dentist–child’ [25] and ‘giving specific instructions’ were the most frequent behaviours [26].

The study has several methodological strengths. Firstly, the present coding scheme acknowledges the core triadic feature of the child dental consultation and covers a wide breadth of verbal and non-verbal behaviours of dental professional, parent and child’s. Specifically, it enables the measurement of observed behaviours in the form of time sequence, duration and frequency, which would be more reliable and ecologically valid than self-reported behaviours [27]. Secondly, as child participation in the medical/dental consultation is so limited and difficult to measure [23], the PaeD-TrICS presents the opportunity to assess child involvement in the consultation. Furthermore, given the research evidence of the benefit of children’s active involvement in their healthcare, the current coding scheme will enable future studies to measure the benefits of young children’s involvement in managing their oral health [28]. Thirdly, the PaeD-TrICS could be applied to examine the effect of dental professionals’ communication and behaviour management strategies and parents’ behaviours in predicting the success of a clinical outcome [28]. Lastly, it could be also used in assessing clinician’s communication skills in triadic consultations involving child patient and parent.

In terms of limitation of the present study, the research team have recognised the potential bias that might have been caused by the presence of the video camera with accompanying researcher. This concern has been assuaged by some empirical evidence [29, 30] that indicated it was acceptable to study healthcare communication involving children using video recording approach. Another limitation is that this coding scheme includes many detailed behaviour codes, where many of them are state-event verbal codes requiring to code the start and end of the duration of each behaviour. This results in a time-consuming coding process for the purpose of accuracy of time registration.

## Conclusion

The PaeD-TrICS is developed and tested with a satisfactory intra- and inter-coder reliability to capture the triadic communication behavioural details in a non-invasive dental consultation setting. It can enhance future research to test the extent of child participation in the healthcare encounter, echoed in the policy of influential bodies: UNICEF and BMA [31, 32].

## Data Availability

The datasets generated and/or analysed during the current study are not publicly available due to the fact that we did not get approval from the ethics committee to provide the raw data of the very young children and their parents who consented to participate in this study. But the aggregate quantitative data and some anonymised quotes are available from the corresponding author on reasonable request.

## Abbreviations

BMA: British Medical Association
EDDN: Extended Duty Dental Nurse
NV: Non-verbal
PaeD-TrICS: The Paediatric Dental Triadic Interaction Coding Scheme
SABICS: The St Andrews Behaviour Interaction Coding Scheme
TSD: Tell-Show-Do
UNICEF: United Nations International Children’s Emergency Fund
